# Uniportal versus multiportal robotic-assisted thoracic surgery pulmonary resections: a propensity score-matched analysis

**DOI:** 10.1186/s12893-025-03221-z

**Published:** 2025-10-09

**Authors:** Ting-Fang Kuo, Shuenn-Wen Kuo, Mong-Wei Lin, Ke-Cheng Chen, Pei-Ming Huang, Jang-Ming Lee

**Affiliations:** 1 Division of Thoracic Surgery, Department of Surgery, National Taiwan University Hospital and National Taiwan University College of Medicine, No. 7, Zhongshan S. Rd., Zhongzheng Dist., 100225 Taipei, Taiwan; 2 Institute of Biomedical Engineering, College of Medicine and College of Engineering, National Taiwan University, No. 1, Sec. 1, Jen-Ai Rd., Zhongzheng Dist., 100233 Taipei, Taiwan

**Keywords:** Minimally invasive surgery, Robotic-assisted surgery, Lung resection surgery, Perioperative outcomes, Pain, Analgesic requirements

## Abstract

**Background:**

Uniportal robotic-assisted thoracic surgery (URATS) has been increasingly adopted in some centers; however, its global acceptance and clinical impact remain uncertain. This study compared the perioperative outcomes of URATS and multiportal robotic-assisted thoracic surgery (MRATS) pulmonary resections.

**Methods:**

Eighteen patients who underwent URATS pulmonary resection between February 2023 and April 2024 were compared with 54 patients who underwent MRATS pulmonary resection between February 2016 and February 2023. Propensity score matching, incorporating age, sex, frailty index, clinical tumor size, nodal stage, operative side, prior treatment, and surgical procedure, was performed to reduce confounding. Perioperative outcomes were analyzed in 18 matched patient pairs.

**Results:**

The URATS group had significantly lower analgesic requirements intraoperatively (12.5 [10.5–13.1] vs. 19 [12.3–21.5] mg; *P =* 0.02) and on the operative day (1.0 [0–3.1] vs. 4.2 [2.0–6.3] mg; *P* = 0.005). They also had shorter intensive care unit stay (0 [0–0] vs. 1 [0–2] day; *P* = 0.03) and postoperative hospital stay (4 [2–7] vs. 7 [5–11] days; *P* = 0.003). However, the docking time was longer in the URATS group than in the MRATS group (11 [8–15] vs. 7 [5–8] min, *P* = 0.006).

**Conclusion:**

URATS appears to be a feasible approach. Lower analgesic requirements in the immediate postoperative period and shorter hospital stays may indicate improved postoperative recovery compared with MRATS.

**Supplementary Information:**

The online version contains supplementary material available at 10.1186/s12893-025-03221-z.

## Introduction

Uniportal video-assisted thoracoscopic surgery (VATS) has been proven safe and feasible in the last two decades. It is widely performed in experienced centers and may have additional benefits for postoperative recovery compared with multiportal VATS [1]. Robotic-assisted thoracic surgery (RATS) features a high-definition optical system that enhances three-dimensional view and offers meticulous instrument control. Even the most complex pulmonary resection procedures can be performed using RATS with outcomes similar to those of open surgery [2, 4]. Previous studies have demonstrated the advantages of RATS over VATS in lymph node dissection and the control of intraoperative bleeding [5, 8]; however, the high costs and 4–5 incisions of traditional multiportal RATS (MRATS) restrict surgeons and patients, making uniportal VATS the mainstay minimally invasive technique in the last decade [9]. With advancements in surgical techniques and technology, the number of incisions required for RATS has gradually decreased. Uniportal RATS (URATS) is now possible and is an important topic in this specialty.

The da Vinci SP system is the most recently designed model for uniportal surgery. Cheng et al. and Lee et al. reported anatomical pulmonary resections through a subcostal incision using this system [10, 12]. However, the system has several practical limitations. The 2.5-cm cannula restricts its use through an intercostal incision, and the subcostal view is not familiar to most thoracic surgeons. In addition, emergent conversion to open thoracotomy can be difficult. The absence of stapling equipment requires support from an experienced assistant, and the system is not yet available in most facilities. In contrast, Gonzalez et al. developed an URATS approach via a traditional intercostal incision using the da Vinci Xi system with robotic staplers [13, 14], while the Shanghai Pulmonary Hospital modified this technique using endoscopic linear staplers [15, 16]. Nonetheless, it remains technically demanding for both the console surgeon and assistant [17]. Despite its potential to combine the ergonomic and oncologic advantages of RATS with the minimal invasiveness of a single incision, URATS adoption faces real-world challenges, including equipment limitations, unfamiliar operative views, a certain learning curve, and unclear advantages or disadvantages compared with the multiportal approach. Our institution modified the URATS approach by Gonzalez et al. and began performing URATS for pulmonary resections in early 2023. Theoretically, fewer incisions may reduce postoperative pain and improve recovery; however, literature comparing URATS and MRATS outcomes is limited [14, 16, 18, 19]. Therefore, we compared the perioperative outcomes of URATS and MRATS pulmonary resections, with a focus on postoperative recovery.

## Methods

### Study population

**Fig. 1:**
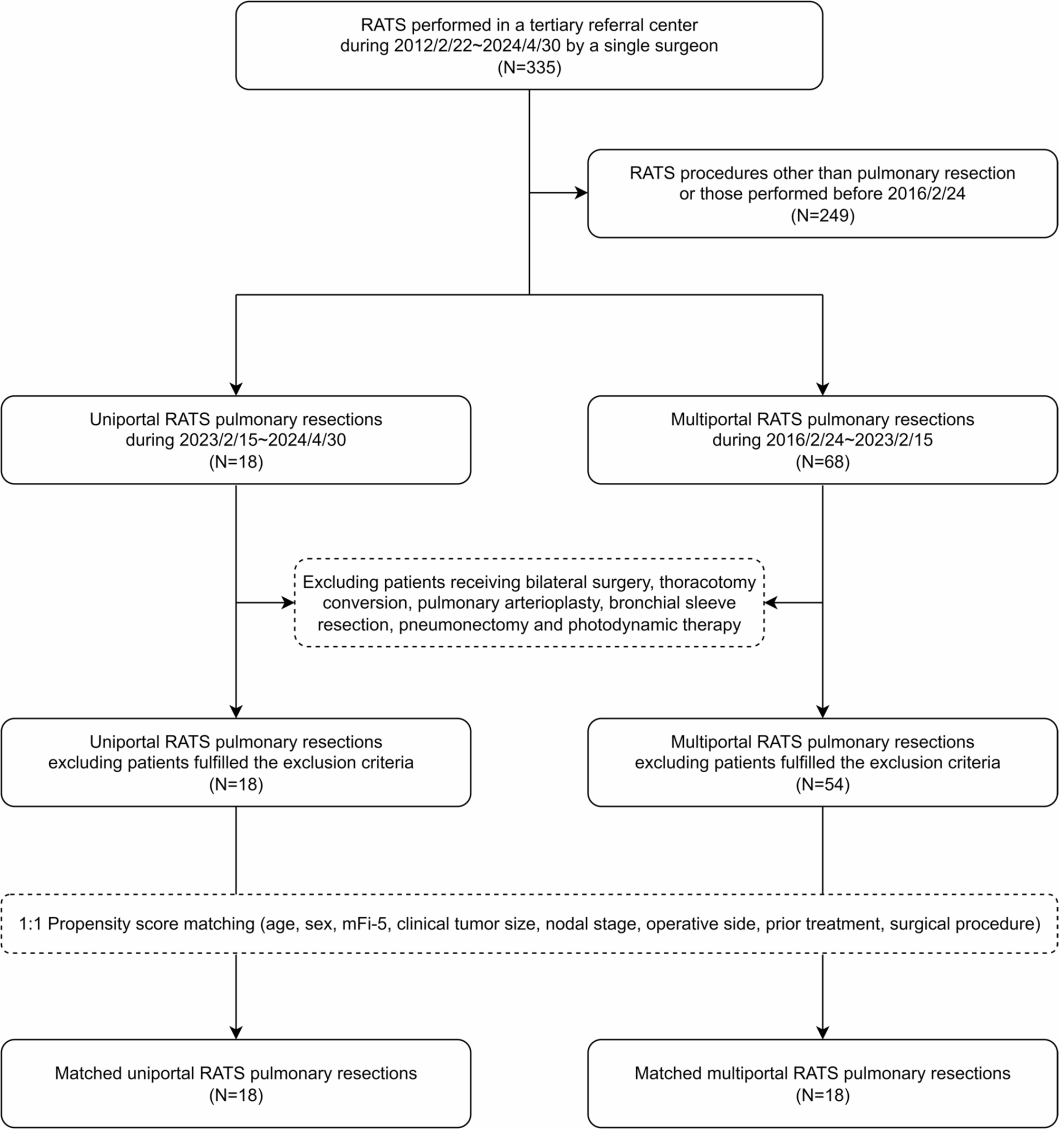
Flowchart of patient inclusion and matching. RATS, robotic-assisted thoracic surgery; mFi-5, five-factor modified frailty index

The flowchart of patient inclusion and matching is shown in Fig. 1. Patients who underwent RATS performed by a single surgeon (JML) since February 2012 were retrospectively reviewed. Pulmonary resections were indicated for resectable pulmonary malignancies and suspected malignant nodules, and these indications were consistent for both URATS and MRATS. URATS for pulmonary resection has been performed since early 2023. Patients receiving URATS pulmonary resection between February 15, 2023, and April 30, 2024, were compared with those treated via MRATS between February 24, 2016, and February 15, 2023. Patients who underwent bilateral surgery, thoracotomy conversion, pulmonary arterioplasty, bronchial sleeve resection, pneumonectomy, or photodynamic therapy were excluded. Ultimately, 18 and 54 patients who underwent URATS and MRATS, respectively, met the inclusion criteria.

### Primary and secondary outcomes

The primary outcome was the length of postoperative hospital stay. The secondary outcomes were postoperative pain score, intraoperative analgesic requirements, analgesic requirements on the operative day, postoperative day 1 (POD1), and postoperative day 2 (POD2), lymph node dissection station, number of lymph nodes dissected, and percentage of nodal upstaging.

### Surgical technique

All procedures were performed when patients received general anesthesia with single-lung ventilation, using either a single-lumen endotracheal tube with an endobronchial blocker or a double-lumen tube. Patients were placed in the lateral decubitus position.

**Fig. 2:**
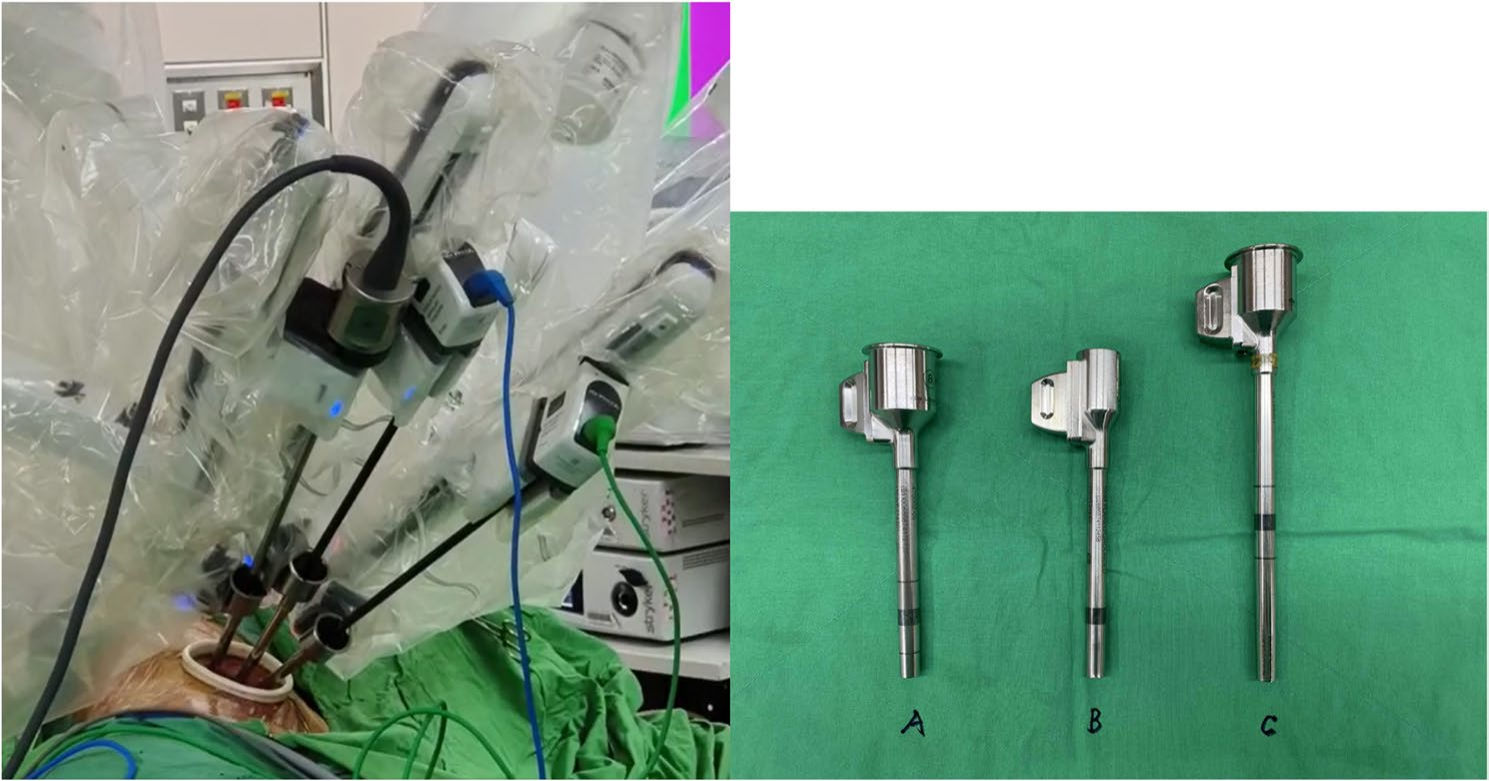
Placement of trocars, **A** regular trocar, **B** flared trocar, **C** long trocar

The URATS procedure, adapted from the technique described by Gonzalez et al., was performed using the da Vinci Xi robotic system (Intuitive Surgical, Inc., Santa Clara, CA, USA) via a single 5-cm incision in the 5th or 6th intercostal space, extending from the midaxillary to the anterior axillary line. A wound protector (Alexis^®^; Applied Medical, Rancho Santa Margarita, CA, USA) was applied after the incision was made. The robotic-assisted system was docked on the cranial and posterior sides of the patient. Arm 1 was cancelled when operating on the right side (arm 2: camera; arm 3: left-hand instrument; and arm 4: right-hand instrument), while arm 4 was cancelled when operating on the left side (arm 1: left-hand instrument; arm 2: right-hand instrument; and arm 3: camera). The right-hand instrument was a Maryland bipolar forceps, and the left-hand instrument was a fenestrated bipolar forceps. The other instruments were monopolar curved scissors, clip appliers, and a needle driver [13]. A long trocar was placed in the middle, whereas two regular or flared trocars were placed bilaterally to improve instrument movement (Fig. 2). Procedures including vascular and bronchial dissection and bronchial anastomosis were performed by the console surgeon, while the endoscopic linear stapler was operated by the assistant, a resident who had assisted in over 50 uniportal VATS procedures, with maximal assistance from the console surgeon. One of the left- or right-hand instruments was dislodged when necessary, primarily to facilitate stapler insertion for vascular, bronchial, or parenchymal division. This was required in approximately 70% of staple firings, amounting to about five times per case, and had minimal impact on operative efficiency [15].

The MRATS procedure, performed using a four-port approach, involved three 1.5-cm incisions at the 7th intercostal space on the midaxillary line, the 5th or 6th intercostal space on the anterior axillary line, and the 8th or 9th intercostal space on the posterior axillary line. An additional 3-cm utility incision was created at the tip of the 9th or 10th rib along the medial margin of the diaphragm to serve as the assistant port for specimen retrieval. Wound protectors (Alexis^®^) were applied to the incisions. The right-hand and left-hand instruments were similar to those of the URATS. The procedures were similar to those of the URATS; however, the endoscopic linear stapler was introduced through the assistant port without dislodging any robotic arm [20].

For patients undergoing bronchoplasty, intraoperative frozen-section analysis was performed to ensure negative bronchial margins. Bronchial anastomosis was performed with either 3–0 barbed absorbable monofilament (V-Loc™; Medtronic, Dublin, Ireland) or 4–0 absorbable polydioxanone (PDS II^®^; Ethicon, Somerville, NJ, USA) continuous running sutures. Intraoperative bronchoscopy was performed to confirm the anastomosis site [20].

Systemic or selective mediastinal lymphadenectomy was performed. The single incision in URATS or the utility incision in MRATS was extended when necessary for specimen retrieval, and any exposed lung parenchyma resulting from fissure dissection was closed with 4–0 polypropylene sutures (PROLENE^®^; Ethicon).

Postoperatively, air leaks were checked using a water-sealing test. The drainless technique was applied to selected patients as described previously; otherwise, a small–medium diameter chest tube was placed via the URATS incision or via the midaxillary incision in MRATS [21].

### Postoperative management

Pain was evaluated using a visual analogue scale every 30 min in the post-anesthesia care unit and every 8 h in the ward [22]. Intravenous (IV) morphine or nalbuphine was administered as needed, and oral celecoxib, acetaminophen, or tramadol was initiated after resuming oral intake. Postoperative chest radiographs were taken on postoperative day 1, and chest tubes were removed in the absence of air leaks and with clinically acceptable drainage output.

### Propensity score matching

Propensity scores were estimated by logistic regression with the surgical approach (uniportal vs. multiportal) as the dependent variable and age, sex, five-factor modified frailty index (mFi-5), clinical tumor size, nodal stage, operative side, prior treatment, and surgical procedure as covariates. We then performed 1:1 greedy nearest-neighbor matching without replacement using a caliper of 0.20. Covariate balance was assessed with absolute standardized mean differences. Although some variables showed residual imbalance, given the small sample and procedural heterogeneity, overall balance improved. To address residual imbalance, we performed covariate-adjusted analyses within the matched cohort (including covariates with post-match absolute standardized mean differences ≥ 0.10 and clustering by matched pairs) as a prespecified sensitivity analysis, which confirmed that the direction and statistical significance of the key outcomes remained unchanged (Supplementary Table S1).

### Data collection

Patient data were retrospectively collected from medical records, including demographic characteristics, surgical procedures, and perioperative outcomes. Frailty, a validated predictor of outcomes after thoracic surgery, was assessed using the mFi-5, comprising diabetes, chronic obstructive pulmonary disease, congestive heart failure, dependent functional status, and hypertension [23]. Prior treatment, such as systemic therapy, radiotherapy, or ipsilateral thoracic surgery, were documented because of their association with intrathoracic adhesions.

Analgesic use was standardized to morphine milligram equivalents. Equivalents included 10 mcg fentanyl (IV), 1 mg nalbuphine (IV), or 12 mg oral tramadol = 1 mg morphine (IV); 2000 mg oral acetaminophen = 3.8 mg morphine (IV); 800 mg oral celecoxib = 4.6 mg morphine (IV) [24, 28]. The highest pain score per day was used as the representative value for the operative day, POD1, and POD2.

### Statistical analysis

Statistical analysis and propensity score matching were conducted using SPSS version 27.0 (IBM Corp., Armonk, NY, USA). Categorical data were presented as numbers (%) and compared using Fisher’s exact or chi-squared tests. Continuous data were reported as means (standard deviation) or medians (interquartile range), with normality assessed using the Shapiro–Wilk test. Student’s t-test was used for normally distributed variables, and the Mann–Whitney U test for non-normal data. Group differences were estimated using the Hodges–Lehmann estimator with 95% confidence intervals (CI). A two-tailed *P* value < 0.05 was considered statistically significant. A post hoc power analysis was performed in the matched sample to assess whether observed effect sizes for key outcomes corresponded to 80% power for large effects.

As an exploratory analysis, the learning curve for docking time in the URATS group was evaluated using the cumulative sum (CUSUM) method. For each case, the deviation of docking time from the group mean was cumulatively plotted, and inflection points in the curve were used to define learning phases.

## Results

A total of 18 patients in the URATS group and 54 in the MRATS group were initially identified. After 1:1 propensity score matching, 18 pairs were included for analysis. Baseline patient characteristics were comparable between the groups, as shown in Table 1.

**Table 1:** Patient characteristics

Variable	URATS group (*N* = 18)	MRATS group (*N* = 18)	*P*-value
Age (years)	62 (11)	68 (11)	0.11^a^
Female sex	13 (72)	7 (39)	0.09^b^
BMI (kg/m^2^)	23.8 (3.6)	23.6 (2.9)	0.86^a^
Past or current smoker	6 (33)	7(39)	1.00^b^
Lung function			
FEV_1_ (% of predicted)	103 (19)	107 (17)	0.48^a^
FVC (% of predicted)	105 (17)	110 (19)	0.46^a^
mFi-5 ≥ 1^e^	8 (44)	10 (56)	0.74^b^
Clinical tumor size (cm)	2.8 (2.1–3.8)	3 (2.4–5.2)	0.36^c^
Clinical nodal stage			0.60^d^
0	17 (94)	16 (88)	
1	1 (6)	1 (6)	
2	0 (0)	1 (6)	
Operative side			0.50^b^
Left	9 (50)	6 (33)	
Right	9 (50)	12 (67)	
Prior treatment^f^	3 (17)	2 (11)	1.00^b^

Clinical tumor size is presented as median (IQR), while other continuous data are presented as mean (SD). Categorical data are presented as number (%)

^a^ Student’s t-test

^b^ Fisher’s exact test

^c^ Mann–Whitney U test

^d^ chi-squared test

^e^ Frailty index including diabetes, chronic obstructive pulmonary disease, congestive heart failure, dependent functional status, and hypertension

^f^ In the URATS group, two patients had prior systemic treatment of metastasis, and one patient had a history of ipsilateral thoracic surgery. In the MRATS group, one patient had prior ipsilateral thoracic surgery and systemic treatment of metastasis, and one patient received neoadjuvant chemoradiation therapy for lung cancer

*BMI* body mass index; *FEV1* forced expiratory volume in the first second; *FVC* forced vital capacity; *IQR* interquartile range; *mFi-5* five-factor modified frailty index; *MRATS* multiportal robotic-assisted thoracic surgery; *SD* standard deviation; *URATS* uniportal robotic-assisted thoracic surgery

**Fig. 3:**
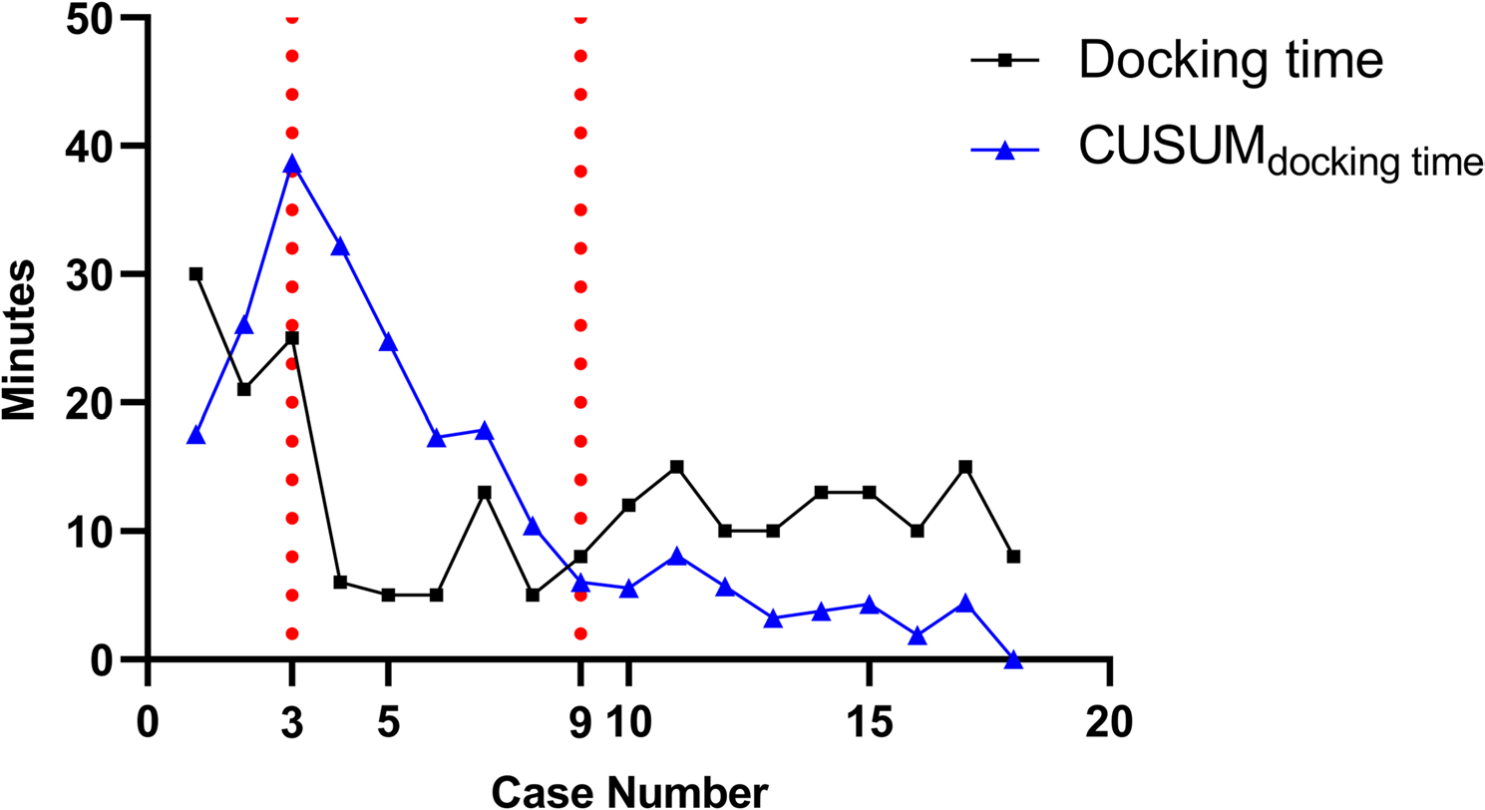
Cumulative sum (CUSUM) learning curve for docking time in the URATS group. Three distinct phases were identified: Learning phase (cases 1–3), characterized by longer docking times; Development phase (cases 4–9), marked by rapid improvement; and Proficiency phase (cases 10–18), where performance stabilized. Vertical dashed lines indicate the transition points between phases

Details of surgical procedures and pathological outcomes are presented in Table 2. No significant differences were observed regarding surgical procedure, number of lymph node stations dissected, lymph node yield, console time, blood loss, proportion of drainless surgeries, pathological findings, pathological stage, or nodal upstaging. Notably, docking time was longer in the URATS group (11 [8 –15] vs. 7 [5–8] minutes; difference [95% CI]: 5 [1 –7] minutes; *P* = 0.006). CUSUM analysis of docking time in the URATS group revealed a triphasic pattern, as illustrated in Fig. 3. Cases 1–3 comprised the Learning phase, characterized by longer docking times; cases 4–9 represented the Development phase, with a rapid reduction in docking time; and cases 10–18 formed the Proficiency phase, where docking time remained stable with minimal fluctuation.

**Table 2:** Robotic-assisted thoracic surgery procedures and pathological outcomes

Variable	URATS group (*N* = 18)	MRATS group (*N* = 18)	*P*-value
Surgical procedure			0.18
Segmentectomy ≤ 2 segments	6 (33)	2 (11)	
Anatomical resection > 2 segments	8 (44)	14 (78)	
Bronchoplastic lobectomy	3 (17)	2 (11)	
Lung and rib resection	1 (6)	0 (0)	
Lymph node retrieval station	5 (3–6)	5 (3–6)	0.78
Lymph node retrieval number	13 (9–21)	15 (11–23)	0.76
Docking time (min)	11 (8–15)	7 (5–8)	0.006^a^
Console time (min)	106 (97–145)	136 (112–181)	0.10
Blood loss (mL)	25 (0–125)	100 (0–185)	0.14
Drainless procedure	5 (28)	4 (22)	1.00
Pathology			0.65
Adenocarcinoma	11 (60)	13 (72)	
Squamous cell carcinoma	1 (6)	2 (11)	
Small cell carcinoma	1 (6)	0 (0)	
Metastasis	2 (11)	2 (11)	
Non-malignancy	3 (17)	1 (6)	
Stage			0.93
I	9 (50)	10 (56)	
II	1 (6)	2 (11)	
III	2 (11)	2 (11)	
IV	1 (6)	1 (6)	
Nodal upstaging	3 (17)	4 (22)	1.00

Continuous data are presented as median (IQR), while categorical data are presented as numbers (%). *P*-values were calculated using the Mann–Whitney U test, Fisher’s exact test, or chi-squared test, as appropriate

^a^ Intergroup difference is 5 (95% Cl: 1 to 7), calculated using the Hodges–Lehmann estimation

*CI* confidence interval; *IQR* interquartile range; *MRATS* multiportal robotic-assisted thoracic surgery; *URATS* uniportal robotic-assisted thoracic surgery

Perioperative outcomes are summarized in Table 3. The intraoperative analgesic requirements (12.5 [10.5–13.1] vs. 19 [12.3–21.5] mg morphine; difference [95% CI]: −6.5 [−9.5 to −1.0]; *P* = 0.02) and analgesic requirements on the operative day (1.0 [0–3.1] vs. 4.2 [2.0–6.3] mg morphine; difference [95% CI]: −2.7 [−4.7 to −0.9]; *P* = 0.005) were lower in the URATS group than in the MRATS group. Morbidity and mortality were not significantly different between the groups; however, one patient in the URATS group died due to pulmonary hypertensive crisis after right upper lobe lobectomy. The URATS group had shorter intensive care unit stay (0 [0–0] vs. 1 [0–2] days; difference [95% CI]: −1 [−2 to 0]; *P* = 0.03) and postoperative hospital stay (4 [2–7] vs. 7 [5–11] days; difference [95% CI]: −3 [−6 to −1]; *P* = 0.003) than the MRATS group.

**Table 3:** Perioperative outcomes

Variable	URATS group *N* = 18	MRATS group *N* = 18	*P*-value
Analgesic requirements			
Intraoperative	12.5 (10.5–13.1)	19.0 (12.3–21.5)	0.02^a^
Operative day	1.0 (0–3.1)	4.2 (2.0–6.3)	0.005^b^
POD1	9.7 (6.1–17.7)	15.1 (9.7–27.7)	0.07
POD2	6.1 (2.9–15.6)	14.3 (6.1–18.0)	0.07
Pain intensity			
Operative day	2 (2–5)	3 (1–4)	0.77
POD1	2 (2–3)	3 (2–4)	0.37
POD2	2 (0–2)	2 (2–2)	0.70
Intravenous analgesia for breakthrough pain	7 (39)	10 (56)	0.50
Morbidity			
Persistent air leakage^c^	2 (11)	1 (6)	1.00
Bronchopleural fistula	0 (0)	0 (0)	
Empyema	0 (0)	0 (0)	
Hemothorax^d^	1 (6)	0 (0)	1.00
Pneumonia^e^	0 (0)	2 (11)	0.48
Mortality	1 (6)	0 (0)	1.00
Intensive care unit stay (days)^f^	0 (0–0)	1 (0–2)	0.03^f^
Time to chest tube removal (days)	1 (0–5)	3 (1–5)	0.22
Postoperative hospital stay (days)	4 (2–7)	7 (5–11)	0.003^g^

Continuous data are presented as median (IQR), while categorical data are presented as number (%). *P*-values were calculated using the Mann–Whitney U test or Fisher’s exact test, as appropriate

^a^ Intergroup difference is −6.5 (95% Cl: −9.5 to −1.0), calculated using the Hodges–Lehmann estimation

^b^ Intergroup difference is −2.7 (95% Cl: −4.7 to −0.9), calculated using the Hodges–Lehmann estimation

^c^ URATS group: one drainless patient developed pneumothorax requiring simple aspiration (Clavien–Dindo IIIa) and one required re-exploration for persistent air leak (IIIb); MRATS group: one drainless patient developed pneumothorax requiring chest tube insertion (IIIa)

^d^ URATS group: one patient developed hemothorax with conservative management (Clavien–Dindo II)

^e^ MRATS group: two patients developed pneumonia treated with medication (Clavien–Dindo II)

^f^ Defined as the number of calendar days from admission to discharge, with stays shorter than 24 h counted as one day. Intergroup difference is −1 (95% Cl: −2 to 0), calculated using the Hodges–Lehmann estimation

^g^ Intergroup difference is −3 (95% Cl: −6 to −1), calculated using the Hodges–Lehmann estimation

*CI* confidence interval; *IQR* interquartile range; *MRATS* multiportal robotic-assisted thoracic surgery; *POD1* postoperative day 1; *POD2* postoperative day 2; *URATS* uniportal robotic-assisted thoracic surgery

## Discussion

We demonstrated shorter intensive care unit and postoperative hospital stays, as well as lower analgesic requirements intraoperatively and on the operative day in the URATS group than in the MRATS group.

Since 2021, when Gonzalez et al. first reported the transcostal URATS using the da Vinci Xi system, many institutions have gradually reduced the number of incisions for RATS and even directly adopted the URATS approach; however, whether the reduction in incisions translates into less postoperative pain and better recovery remains unclear. The quality of resection surgery for pulmonary malignancies is also a concern.

An unmatched comparison between URATS and biportal RATS has previously shown lower postoperative pain scores and shorter hospital stays in the URATS group [13, 16]. The pain score on the visual analogue scale is the most commonly used tool for pain evaluation because it is simple and rapid; however, it is subjective and reflects pain intensity only at a single time point. In our study, to provide a more standardized measure, analgesic requirements were converted to morphine milligram equivalents using standard conversion factors, consistent with recent literature, and were evaluated alongside the visual analogue scale [29, 30]. Pain scores and the proportion of rescue analgesics for breakthrough pain were comparable, indicating that analgesic use was appropriately adjusted to baseline pain in both groups. Nevertheless, the URATS group had significantly lower analgesic requirements intraoperatively and on the operative day, possibly because pain was most pronounced during intraoperative manipulation through the incisions. As postoperative pain improved over time, differences between uniportal and multiportal approaches became less apparent. However, the early reduction in analgesic requirements suggests that URATS provides better recovery in the immediate postoperative period than MRATS. Regarding the quality of pulmonary resection for malignancy, the number of lymph node stations dissected, lymph node yield, and the nodal upstaging rate were comparable between the two groups. This is similar to a previous comparison between uniportal and multiportal VATS [1].

Docking time was longer in the URATS group than in the MRATS group, reflecting the early learning curve of adapting three robotic arms through a single incision. CUSUM analysis confirmed a triphasic adaptation with stabilization after about 10 cases, consistent with previous reports [14]. By contrast, console time was comparable between groups. The operating surgeon had completed more than 300 MRATS cases and had proficiency in uniportal VATS. Thus, the general RATS learning curve had already been surpassed and facilitating a smooth transition to URATS [31]. This likely minimized differences in console performance despite the technical challenges of a single incision.

In our URATS approach, the same robotic platform and instruments as in MRATS were used, with comparable equipment availability and procedural costs. The main technical challenge is placing all instruments through a single incision while minimizing collisions, which requires surgeons to adapt to uniportal hand positioning and approach angles, with greater reliance on the assistant for exposure. Hardware adjustments, such as offsetting or downsizing trocar heads, can further reduce arm collisions (Fig. 2) [32]. However, in hospitals without established robotic programs, the high initial investment and maintenance costs may still limit adoption. Moreover, while surgeons and assistants experienced in uniportal VATS can adapt relatively quickly, such teams remain uncommon, and limited training opportunities together with sparse literature may further deter MRATS-trained surgeons from transitioning to URATS.

This study had several limitations. First, the small sample size limited generalizability and statistical power. Post hoc analysis suggested adequate power to detect large effects for recovery outcomes, but insufficient power for rare events such as morbidity and mortality. Second, the retrospective design and use of historical controls may have introduced bias; although we used propensity score matching and prespecified covariate-adjusted sensitivity analyses within the matched cohort, residual confounding and subtle, unmeasured changes in perioperative care cannot be excluded. Third, the use of morphine milligram equivalent conversion standardized dosing across routes but did not capture interindividual variability. Finally, the single-center design with an experienced surgeon–assistant team may limit the applicability of our findings to other settings. Larger prospective trials are warranted to confirm these findings.

In conclusion, URATS appears to be a feasible approach. Lower analgesic requirements in the immediate postoperative period and shorter hospital stays may indicate improved postoperative recovery compared with MRATS.

## Supplementary Information

Supplementary Material 1

## Data Availability

The authors confirm that the data generated and analyzed during this study and the raw data are available from the corresponding author, upon reasonable request.
