# Supplementary material for: Uniportal versus multiportal robotic-assisted thoracic surgery pulmonary resections: a propensity score-matched analysis
Source: BMC Surg. 2025 Oct 9;25:465. doi: 10.1186/s12893-025-03221-z (PMC12512549; doi:10.1186/s12893-025-03221-z)
Supplement: Supplementary file 1 — Supplementary Material 1 [file 12893_2025_3221_MOESM1_ESM.docx]

**Supplementary Table S1.** Absolute standardized mean differences of covariates after propensity score matching

| **Variable** | **Absolute SMD^a^** |
| --- | --- |
| Age (years) | 0.55 |
| Sex |  |
| Male | 0.67 |
| Female | 0.67 |
| mFi-5^b^ |  |
| 0 | 0.22 |
| 1 | 0.00 |
| 2 | 0.16 |
| 3 | 0.34 |
| Clinical tumor size (cm) | 0.35 |
| Clinical nodal stage |  |
| 0 | 0.20 |
| 1 | 0.00 |
| 2 | 0.34 |
| Operative side |  |
| Left | 0.34 |
| Right | 0.34 |
| Prior treatment |  |
| No | 0.16 |
| Yes | 0.16 |
| Surgical procedure |  |
| Segmentectomy ≤ 2 segments | 0.54 |
| Anatomical resection > 2 segments | 0.68 |
| Bronchoplastic lobectomy | 0.16 |
| Lung and rib resection | 0.34 |

^a^ Sensitivity analysis was performed using covariate-adjusted regression models within the matched cohort, including covariates with post-match |SMD| ≥0.10, with standard errors clustered by matched pairs. Results were consistent with the main analysis.

^b^ Frailty index including diabetes, chronic obstructive pulmonary disease, congestive heart failure, dependent functional status, and hypertension.

SMD, standardized mean differences
